# Supplementary material for: Active ballistic orbital transport in Ni/Pt heterostructure
Source: Nat Commun. 2024 May 29;15:4568. doi: 10.1038/s41467-024-48891-0 (PMC11137139; doi:10.1038/s41467-024-48891-0)
Supplement: Supplementary file 1 — Supplementary Information [file 41467_2024_48891_MOESM1_ESM.pdf]

Active Ballistic Orbital Transport in Ni/Pt Heterostructure

Sobhan Subhra Mishra[1,2], James Lourembam[3], Dennis Jing Xiong Lin[3], Ranjan Singh[1,2]*

[1]Division of Physics and Applied Physics, School of Physical and Mathematical Sciences, Nanyang Technological University, Singapore 637371
[2]Centre for Disruptive Photonic Technologies, The Photonics Institute, Nanyang Technological University, Singapore 639798
[3]Institute of Materials Research and Engineering, Agency for Science, Technology and Research, 2 Fusionopolis Way, Singapore 138364

* *Corresponding Author- ranjans@ntu.edu.sg*

Supplementary Information

Section S1: Terahertz emission spectroscopy setup

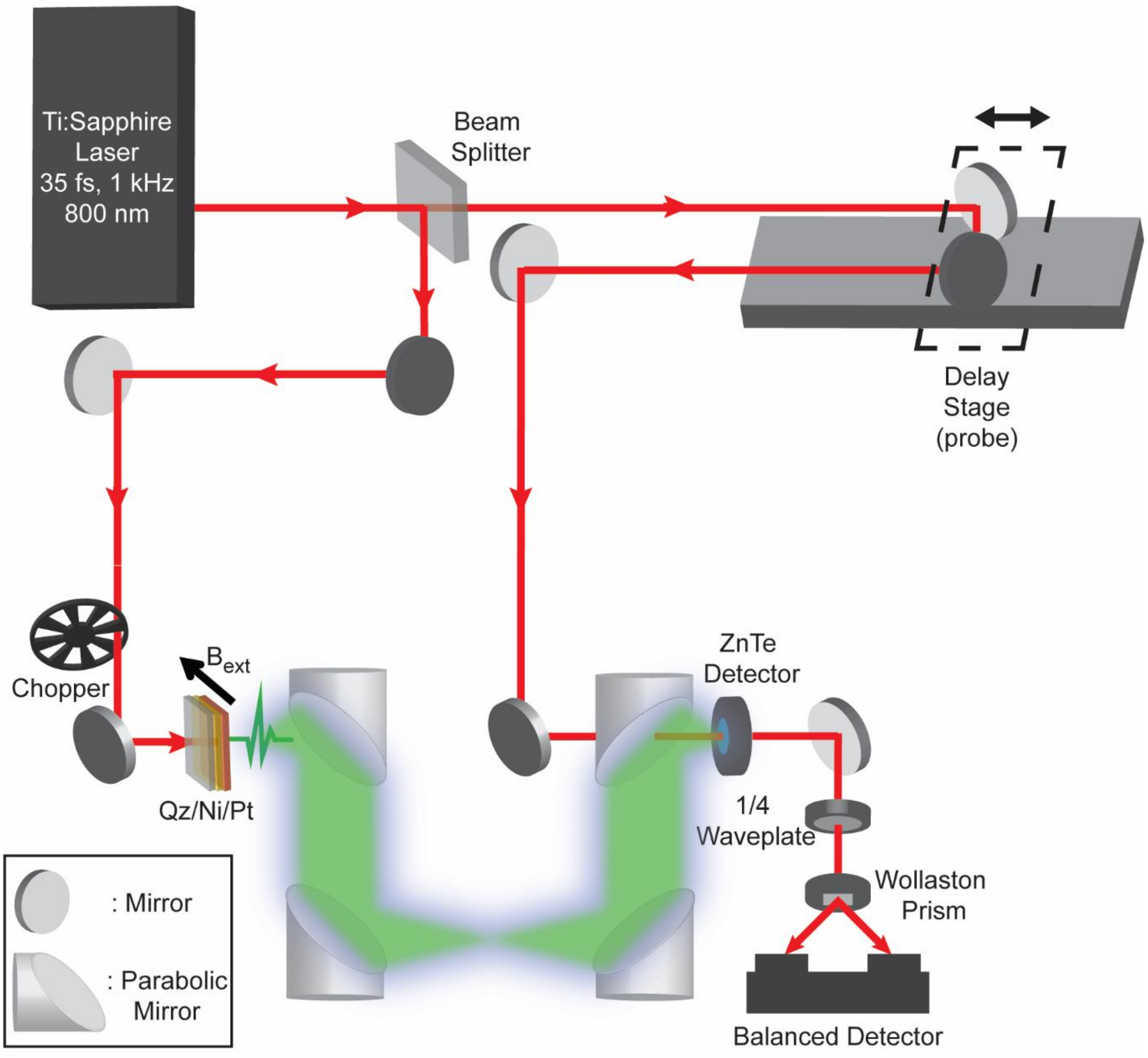

Figure S1: THz emission spectroscopy set up

Fig S1 shows the complete set up for the THz pulse generation. Ultrafast photoexcitation of Orbitronic THz emitter leads to emission of THz radiation which is collected by parabolic mirror and detected by a 1 mm ZnTe <110> crystal. A time matched probe beam is used to detect the signal through electro optic sampling. Details of the THz detection scheme is given in the method section of the manuscript.

Section S2: Terahertz emission from different Orbitronic sample [Ni (3 nm)/Ru (3 nm)]

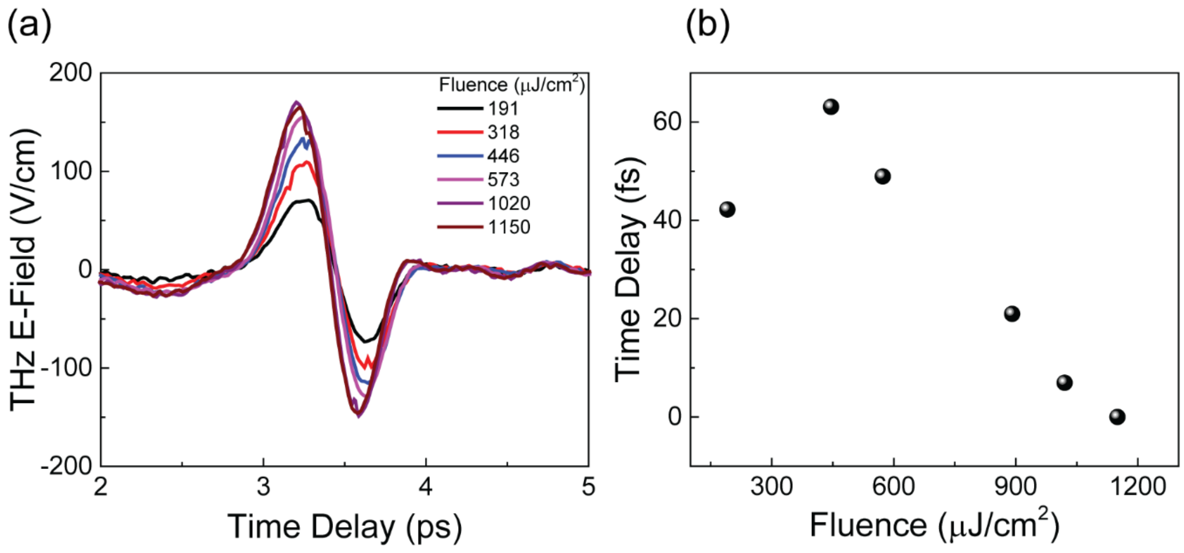

Figure S2: Fluence dependent THz emission from Ni (3nm)/ Ru (3 nm) (a) Emitted THz from Ni (3 nm)/ Ru (3 nm) at different fluence (b) Time delay for different fluences; Initially the shift is towards right indicating the decrease in orbital velocity and after a critical fluence, delay starts decreasing with increase in fluence showing swifter orbital transport

Fig S2 shows the THz emission from Ni (3nm)/ Ru (3 nm) at different fluences. A trend similar to Ni/Pt is observed as mentioned in main manuscript. Initially a delayed emission of THz radiation was observed as we increase the fluence till a critical fluence after which there was a left shift of THz pulses indicating a swifter orbital current.

Section S3: Delay calculation due to the thickness of Pt

Previous calculation has proved that beyond 4 nm there is no spin to charge conversion in Pt. So, any delay in spin transport-based THz emission system would be because of the thickness of Pt[1]. To prove that the delay in the emitted THz in Ni/Pt as we increase the thickness of Pt is due to the long relaxation of Orbital current, we have calculated the possible delay in THz pulse due to the thickness of Pt below.

Experimentally it has been measured that the refractive index of Pt[2] in THz from 0.1 to 3 THz is n ~ 80

Extra thickness of Pt THz needs to cover for Ni (3 nm)/ Pt (x nm) where x = 6, 9, 18 w.r.t Ni (3 nm) / Pt (3 nm) = (x-3) nm

So, time delay expected = $\frac{(x-3)nm}{c/n}$ *(S1)*

Here c = speed of light in vacuum

For x = 6, time delay ~ 0.8 fs

x = 9, time delay ~ 1.6 fs

x = 18, time delay ~ 4 fs

This proves that the delay seen in the system is because of the long relaxation of the orbital of the orbital transport.

Section S4: Rough extraction of Orbital relaxation length

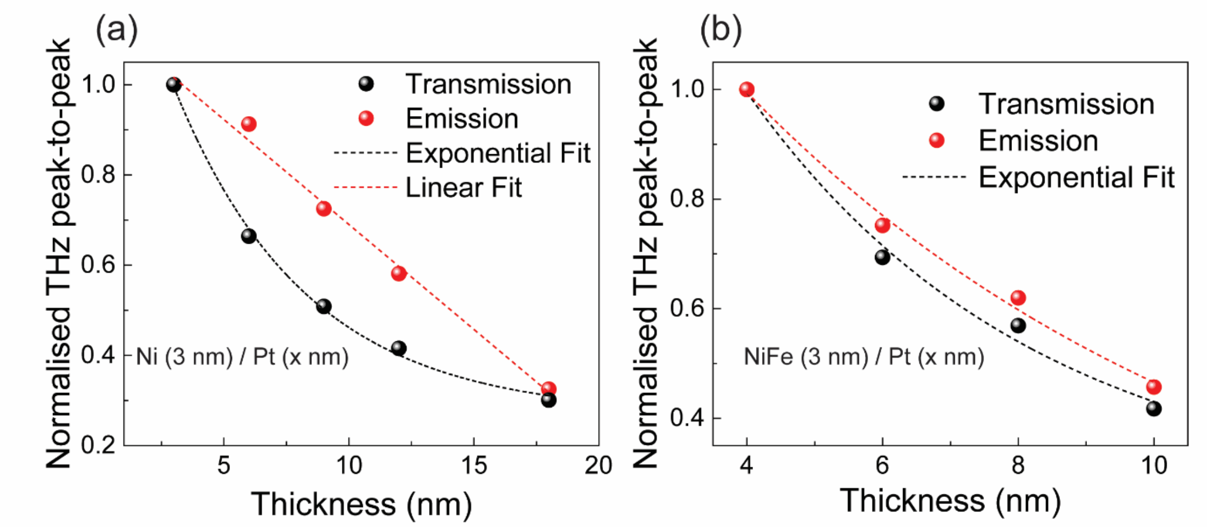

Figure S3: Comparison of THz transmission and THz emission for (a) Ni (3 nm) / Pt (x nm) showing a linear decay in the emitted THz pulse in contrast to an exponential decay in the transmitted THz pulse indicating a longer transport phenomena (b) NiFe (3 nm)/ Pt (x nm) showing an exponential decay in both emitted and transmitted THz pulse indicating a shorter transport phenomenon.

It is anticipated that, beyond the relaxation length, both THz transmission and THz emission will display similar patterns with increasing thickness, coinciding with each other. Figure S3 depicts a comparison between THz transmission and emission for Ni/Pt and NiFe/Pt. In the instance of Ni/Pt (*x* nm), the peak-to-peak THz pulse emitted shows a linear decrease, indicating dispersion and attenuation, contrasting with the exponentially decreasing transmitted peak-to-peak THz pulse, until they coincide at a thickness of 18 nm, proving a relaxation length of greater than 18 nm. However, for NiFe/Pt, both the transmitted and emitted pulses exponentially decrease with increasing thickness, and the normalized amplitudes for both emission and transmission almost coincide. The examination of THz transmission and emission, alongside the delay in the emitted pulse, offers evidence that the emitted beam stems from long-range transport in Pt in the case of Ni/Pt.

Section S5: Terahertz transmission spectroscopy of Ni (3 nm) / Pt (x nm)

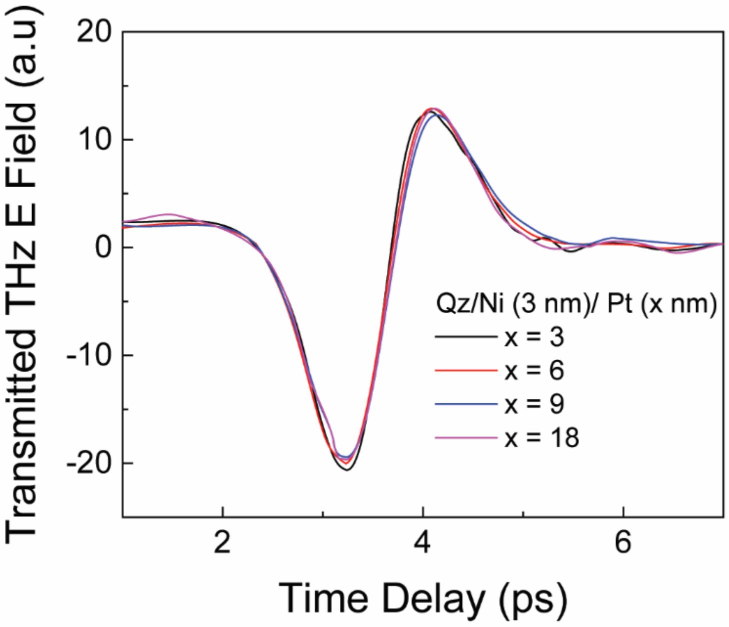

Figure S4: Transmitted THz E-Field from Ni (3nm) Pt (*x* nm) where *x* = 3, 6, 9, 18 normalized to the transmission in Ni (3 nm) / Pt (3 nm) indicating no delay or chirping in the transmitted pulse.

THz transmission measurement of Ni (3 nm)/ Pt (x nm) with varying x is performed. As seen in Figure S4, the transmitted THz pulses neither have a delay nor a pulse chirping when we pass THz through the samples. This proves that the variation in the thickness of the substrates used is negligible and does not affect the THz emitted and therefore does not contribute to the delay observed. Additionally, this also proves that the delay and chirping seen in the emitted pulse cannot be due to the refractive index of Pt.

Section S6: Fluence dependent study of NiFe (3 nm)/Pt (*x* nm)

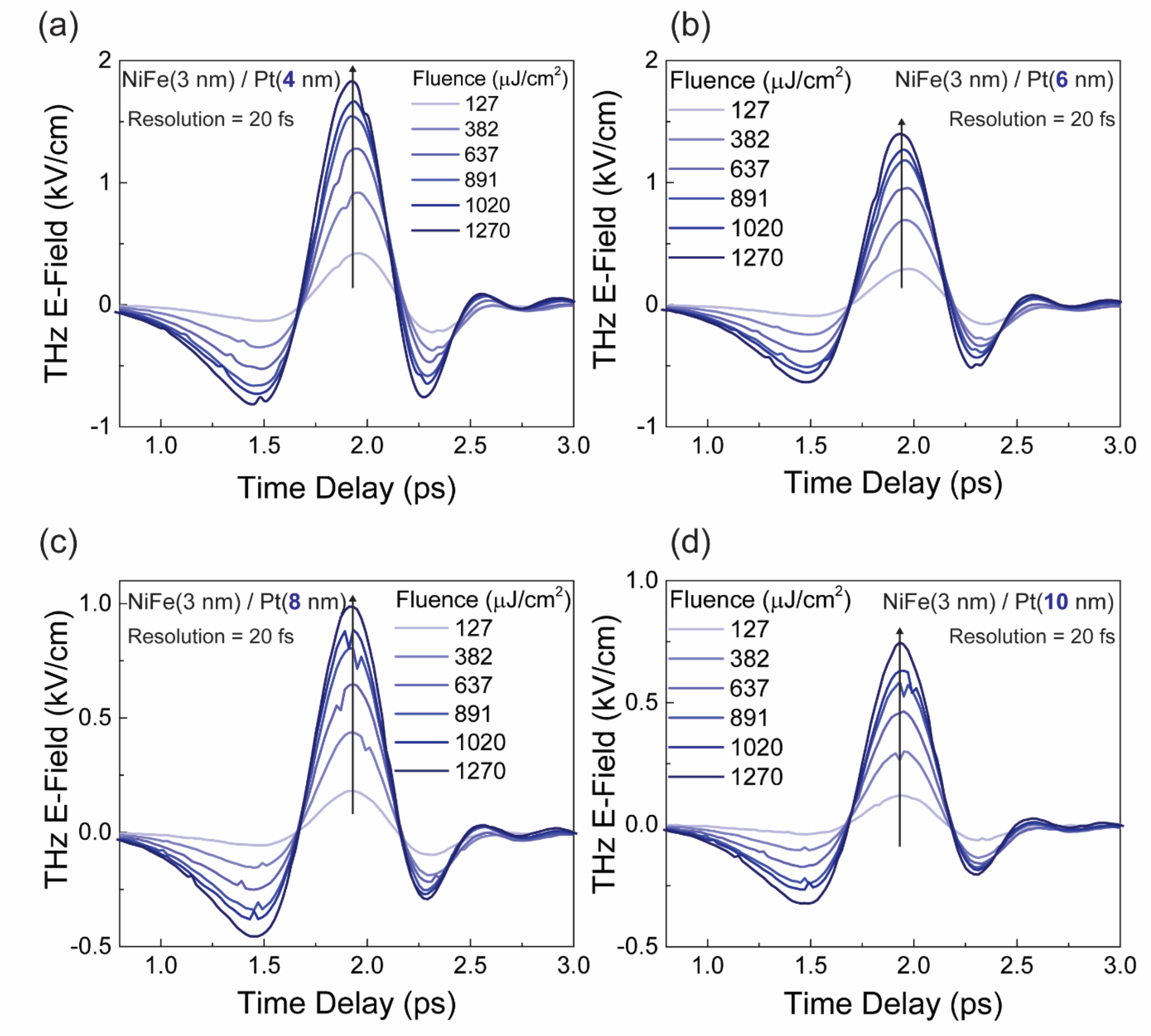

Figure S5: Fluence Dependent Study of THz emission from NiFe (3 nm)/ Pt (*x* nm) when (a) x = 4 nm, (b) x = 6 nm, (c) x = 8 nm, (d) x = 10 nm indicating no shift in the emitted pulse with increase in laser fluence

Section S7: Delay due to change in optical density of the filter

The laser fluence was controlled by a linear ND filter. Before the emitter a linear ND filter was placed, and it was moved by a mechanical translation stage to change the applied fluence.

To ensure that the THz shift is not because of the change in optical density of the ND filter, two experiments were conducted

1. The same filter was put in front of a 1 mm ZnTe detector and the delay in the detected THz pulse emitted from a LN crystal was monitored. By changing the position of the filter, probe power of the detecting the emitted pulse was changed. The detected THz signal shows no sign of shift as we change the probe power indicating that no temporal shift is introduced because of the change in optical density of the filter as clearly seen in Figure S6(a).
2. As another test, the filter was put in front of a LN emitter and the possible delay in the emitted THz by changing the pump power was monitored. As seen in Figure S6(b), there is no shift in the emitted THz pulse.

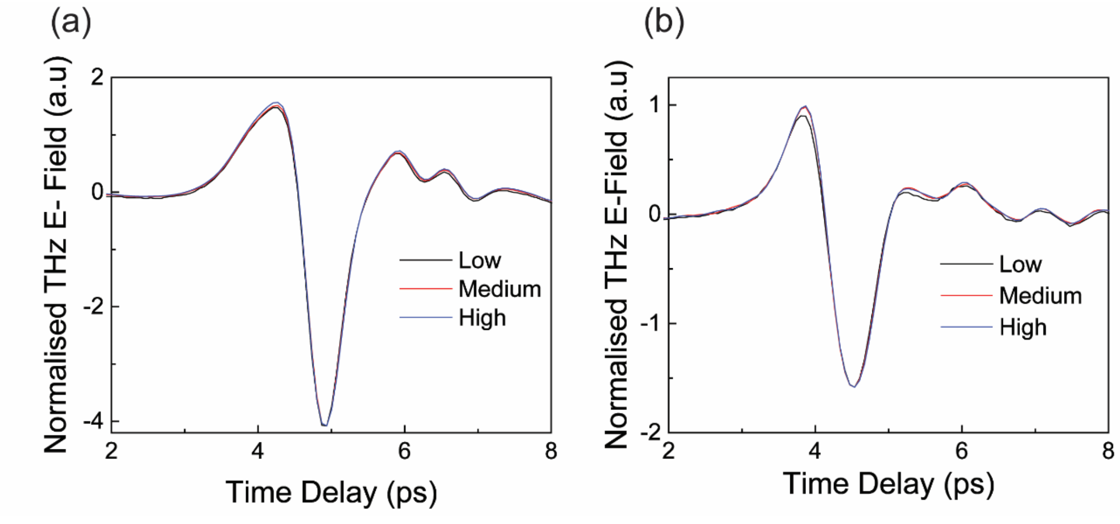

Figure S6: THz recorded from LN emitter by changing the position of the Linear ND Filter (a) When the filter is in front of the detector (b) When the filter is in front of the emitter Both indicate no shift in the THz pulse eliminating the effect of optical density of the filter

Section S8: Error calculations

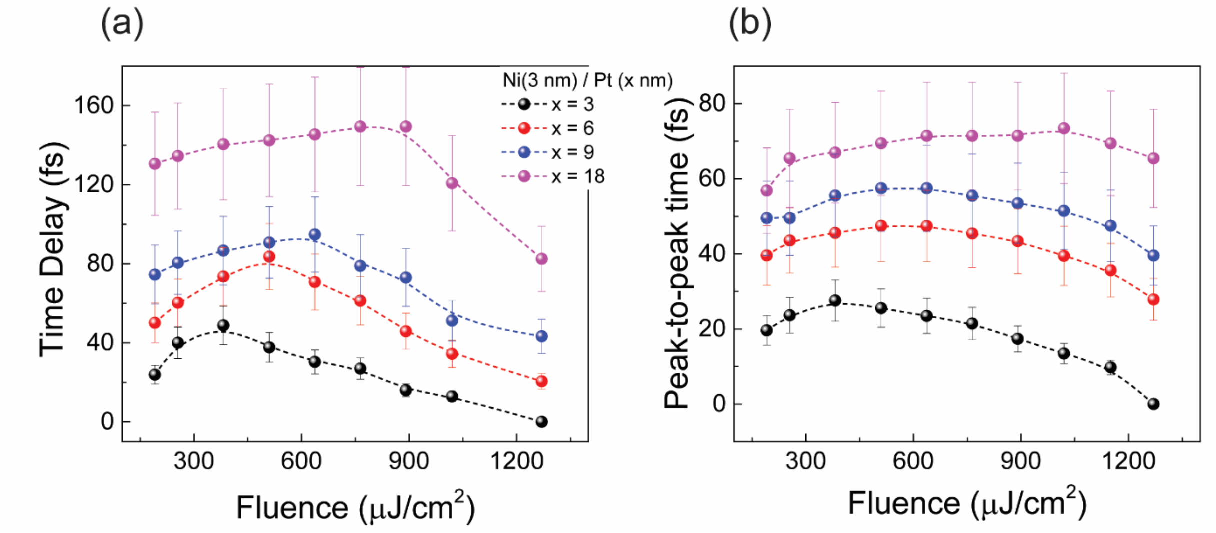

Figure S7: Active Control of Ballistic Orbital Transport (a) Time delay for different fluence is shown for Ni (3 nm)/ Pt (x nm) with x = 3, 6, 9, 18. Initially the shift is towards right indicating the decrease in orbital velocity and after a critical fluence, delay starts decreasing (b) THz pulse width for different fluence is shown for Ni (3 nm)/ Pt (x nm) with x = 3, 6, 9, 18. Initially the shift is towards right indicating the decrease in orbital velocity and after a critical fluence, delay starts decreasing.

Section S9: Backlash error of the delay stage

The measurement setup involves the utilization of the PI linear delay stage VT-80 (Model no 62309250-0000), having a backlash error of 0 and bidirectional repeatability of 10 microns, as specified in the reference[3]. However, to ensure the potential backlash error inherent in the delay stage, monodirectional scanning is conducted exclusively.

In this approach the delay stage is scanned in a single direction to mitigate any potential backlash issues. The monodirectional repeatability of our delay stage is 0.8 microns[3]. This strategy allows the replication of the emitted THz pulses during repeated scans, as illustrated in Figure S8.

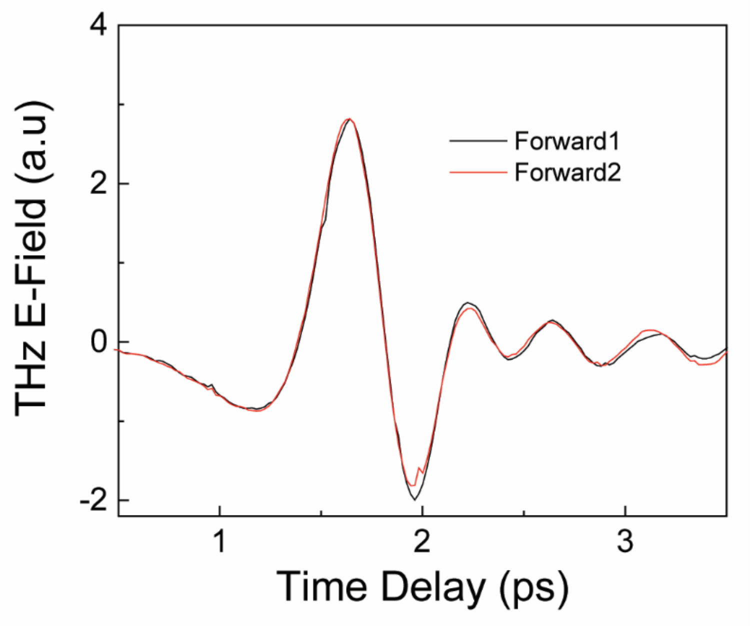

Figure S8: THz emitted from Ni (3 nm)/Pt (3 nm) with 2 forward scans indicating negligible effect of the backlash error of the delay stage

Section S10: THz emission and transmission in Ni (3 nm)/ Ru (x nm)

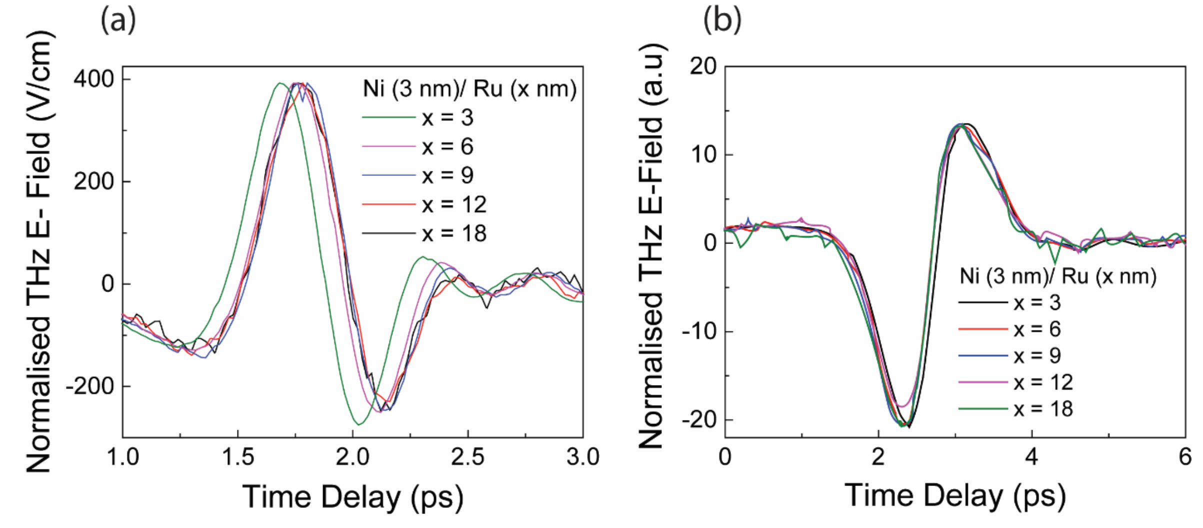

Figure S9: THz measurement of Ni (3 nm) / Ru (*x* nm) where *x* = 3, 6, 9, 12, 18 (a) Terahertz emission measurements. The delay in the emitted pulse is absent beyond 6 nm indicating an orbital relaxation length of <6 nm. (b) Terahertz Transmission measurements

Figure S9 shows the THz emission and transmission from Ni (3nm)/ Ru (*x* nm) with *x* = 3, 6, 9, 12, 18. Previously experimentally it is verified that the orbital diffusion length of Ru is

around 3.8 nm[4]. Therefore, as the thickness of Ru is increased from 3 nm to 6 nm, there is a considerable shift in the emitted pulse. However, as we increase the thickness further, the delay is not observable. This also proves that the delay in Ni/Pt (*x* nm) is because of the long transport entity travelling in Pt.

References

1. Agarwal, P. *et al.* Secondary Spin Current Driven Efficient THz Spintronic Emitters. *Adv. Opt. Mater.* 2301027 (2023) doi:10.1002/adom.202301027.
2. Yasuda, H. Measurement of Terahertz refractive index of metals. in *2008 Asia-Pacific Microwave Conference* 1–4 (IEEE, Macau, 2008). doi:10.1109/APMC.2008.4958133.
3. VT-80 Linear Stage. https://www.physikinstrumente.com/en/products/linear-stages/stages-with-stepper-dc-brushless-dc-bldc-motors/vt-80-linear-stage-1206300.
4. Santos, E. *et al.* Exploring inverse orbital Hall and orbital Rashba effects: unveiling the oxidation states of the Cu surface. (2024) doi:10.48550/ARXIV.2402.00297.
