# Supplementary material for: Active ballistic orbital transport in Ni/Pt heterostructure
Source: Nat Commun. 2024 May 29;15:4568. doi: 10.1038/s41467-024-48891-0 (PMC11137139; doi:10.1038/s41467-024-48891-0)
Supplement: Supplementary file 3 — Reporting Summary [file 41467_2024_48891_MOESM3_ESM.pdf]

nature research

Corresponding author(s): Ranjan Singh

Lasing Reporting Summary

Nature Research wishes to improve the reproducibility of the work that we publish. This form is intended for publication with all accepted papers reporting claims of lasing and provides structure for consistency and transparency in reporting. Some list items might not apply to an individual manuscript, but all fields must be completed for clarity.

For further information on Nature Research policies, including our data availability policy, see Authors & Referees.

Experimental design

Please check: are the following details reported in the manuscript?

1. Threshold

   Plots of device output power versus pump power over a wide range of values indicating a clear threshold

   ☐ Yes ☒ No — We are not claiming any lasing. Our work is based on THz emission

2. Linewidth narrowing

   Plots of spectral power density for the emission at pump powers below, around, and above the lasing threshold, indicating a clear linewidth narrowing at threshold

   ☐ Yes ☒ No — We are not claiming any lasing. Our work is based on THz emission

   Resolution of the spectrometer used to make spectral measurements

   ☐ Yes ☒ No — We are not claiming any lasing. Our work is based on THz emission

3. Coherent emission

   Measurements of the coherence and/or polarization of the emission

   ☐ Yes ☒ No — We are not claiming any lasing. Our work is based on THz emission, so we are not calculating polarization

4. Beam spatial profile

   Image and/or measurement of the spatial shape and profile of the emission, showing a well-defined beam above threshold

   ☐ Yes ☒ No — We are not claiming any lasing. Our work is based on THz emission

5. Operating conditions

   Description of the laser and pumping conditions
   Continuous-wave, pulsed, temperature of operation

   ☒ Yes ☐ No — The description of laser that we are using to pump our THz emitter has been given in page 17 under the method section of our main manuscript file.

   Threshold values provided as density values (e.g. W cm^{-2} or J cm^{-2}) taking into account the area of the device

   ☐ Yes ☒ No — We are not claiming any lasing. Our work is based on THz emission

6. Alternative explanations

   Reasoning as to why alternative explanations have been ruled out as responsible for the emission characteristics
   e.g. amplified spontaneous, directional scattering; modification of fluorescence spectrum by the cavity

   ☐ Yes ☒ No — We are not claiming any lasing. Our work is based on THz emission

7. Theoretical analysis

   Theoretical analysis that ensures that the experimental values measured are realistic and reasonable
   e.g. laser threshold, linewidth, cavity gain-loss, efficiency

   ☐ Yes ☒ No — We are not claiming any lasing. Our work is based on THz emission

8. Statistics

   Number of devices fabricated and tested

   ☐ Yes ☒ No — We are not claiming any lasing. Our work is based on THz emission

   Statistical analysis of the device performance and lifetime (time to failure)

   ☐ Yes ☒ No — We are not claiming any lasing. Our work is based on THz emission
